# Progress in Medicine

**Published:** 1897-07-10

**Authors:** 


### Progress in Medicine.

### DISEASES OF THE DIGESTIVE ORGANS.

(*Continued from page* 232.)

**ntestines.**—Dieulafoy[1] describes a very little known disorder, which he terms intestinal gravel, and which appears to be connected with gout and lithiasis. The symptoms include pain, sometimes so severe as to simulate biliary colic or appendicitis. The pain is not localised in one spot, but is accompanied by tympanites and followed by solid or liquid stools containing gravel mixed with mucus. Several attacks of pain occur in the twenty-four hours before the gravel is expelled, and may recur at intervals for years. The stools should be carefully examined, and when the gravel is found treatment for gout with Vichy water and a regulated diet are indicated. The gravel contains calcium and magnesium salts, with a trace of silica, but no cholesterin.

P. F. Macgregor[2] discusses the diagnosis of duodenal ulcer, and emphasises the latency of the symptoms, the long time, half an hour to two hours after food, when pain comes on, and the lighter colour of the stools compared to those in gastric ulcer, where the blood is, of course, mixed with gastric juice. He gives an account of a case in a patient, aged 45, who, after a month or two of vague abdominal pains, passed a dark-coloured stool, and next day vomited a quantity of dark blood, some of the clots being of a sausage shape. After treatment by ice and rectal feeding she recovered, but suffered from another attack 12 months later.

The gases in the intestine are derived either from food stuffs or from air swallowed, or by secretion from the blood. H. Muir Evans[3] notices the large amount of nitrogen and of carbonic acid present in gases from the intestines, even if we put aside those cases where sulphuretted hydrogen and marsh gas, which is formed from leguminous food, are produced. He believes it probable that in many cases the gas is secreted by the mucous membranes. In fishes there is a physiological secretion of gas in the air bladder, which in some cases has no connection with the intestines. In that of the codfish he found the gas to consist of oxygen 15 per cent. and nitrogen 85 per cent., which gives more nitrogen than exists in air, and much more than is found in the air held in solution by the water where the fish swims. In the blood of mammals the proportion of nitrogen in the blood is very small indeed, and as it exists in intestinal flatus in much greater proportion than any other gas, he thinks that it is produced by an active metabolic process, and is not merely atmospheric air from which the oxygen has been removed.

The editor of the *Therapeutic Gazette*,[4] in reviewing a proposed method of treatment for intussusception without operation, recommends irrigation by water while the patient is anæsthetised and placed face downwards with the hips raised, but he insists that the water should be allowed to enter the bowel slowly with no greater pressure than that of a column eighteen inches or two feet above the abdomen. Any greater pressure may rupture the bowel, but he has proved by experiment that the above pressure is sufficient to send a stream through the colon, cæcum, and small intestines, and out through the œsophagus. If treatment by irrigation is attempted at all the precautions mentioned above are most important. We are rarely, if ever, troubled here by the ankylostoma duodenale, though it is a terrible scourge in the West Indies and other tropical countries, causing a fatal form of anæmia. Hence great importance is attached to the discovery of a remedy, thymol, which acts as a specific. Otho Galgey,[5] after trying in vain various other substances at St. Lucia, reports most successful results in about eighty cases treated with thymol. The patients are kept on milk diet for a day or two, a brisk purgative is given, and the next day two doses of thymol of 15 or 20 grains are administered, with two hours interval. If necessary, a final dose of castor oil is taken, and rapid recovery follows. Zinn and Jacobi,[6] however, believe that the ankylostoma occurs in great numbers of negroes without producing any symptoms. Indeed, their intestinal canal contains many other parasites. The ankylostoma produces a toxin destructive of red blood cells, as well as abstracting large quantities of blood, and it is possible that the negro has developed some special immunity against it. The patients whom Galgey treated were Coolies and Creoles, and in Egypt, of course, a large part of the population, subject to its ravages, have little or no negro blood.

**Appendicitis.**—Goodhart[7] notices a class of cases to which the term "appendicular colic" is not inapplicable. In them the appendix is not a healthy one striving to expel a foreign body; there is usually disease in the mucous membrane, and usually a stricture, but, though there may be recurrent attacks and considerable danger, no inflammatory changes external to the appendix are found. He goes on to remind his hearers that among the symptoms of appendicitis it is important to remember that purging may take the place of constipation, that the temperature may be normal, and that stone in the kidney or gall bladder, or ovarian disease may produce a train of symptoms almost indistinguishable from those due to the appendix. Again, the abscesses starting from it may be diagnosed as the results of spinal caries, of liver disease, or of tubercular peritonitis. In relapsing cases he takes as indications of an abscess formation, a thickly coated dry tongue, the absence of improvement, while the fever and local tenderness and swelling remain unaltered on the fourth or fifth day. Apart from exceptional cases, this is most commonly the date when operative measures are desirable. Finally, it occasionally happens that where general peritonitis has been diagnosed from tympanites, there is merely an underlying abscess which has pushed up the intestines in front of it. Goluboff[8] holds that appendicitis is an epidemic infectious disease. He shows how it occurs plentifully in certain streets and at certain seasons, and occasionally in those who have associated with the sick. In origin and spread it is closely akin to dysentery, while polyarthritis is at times a complication of both diseases. Charrin[9] found a peculiar form of appendicitis common in the animals kept in a certain laboratory, and he isolated a particularly virulent form of streptococcus which he thinks may be the cause. Josué[10] produced the lesions of appendicitis by the intravenous injection of this very microbe, or of fæcal matter, and in his paper refers to the instances of hæmorrhagic appendicitis found in cases of infection by Friedlander's bacillus and phosphorous poisoning.

Talamon[11] actually declares that the mortality after operation is greater than without it, even when we take account of the fact that the severest cases are not left to medical treatment. He insists that the clinical aspect of each case must be considered before we recommend operation. Out of 64 acute cases he has attended since 1890, he states that 30 were plastic peritonitis, 13 simple parietal appendicitis, 7 diffuse and 14 local suppurative peritonitis, so that he considers that two-thirds of all acute cases are not suitable for surgical intervention. In MacBurney's 14 cases out of 24 of diffuse peritonitis which recovered after incision and lavage, it is not clear that the peritonitis was general in any which recovered. Hence Talamon regards operations here as useless. The diagnosis of this form is, however, difficult; the rigidity of the abdomen and the distension are not altogether to be relied on, but a small weak pulse above 120 in an adult is of great importance. If, then, there is intense peritoneal inflammation, operation is better delayed; but if local suppuration is clear, surgical aid should be demanded before the fever of suppuration sets in.

Renvers, of Berlin,[12] reports a mortality of 9·7 per cent. on all cases, which rose in those operated on to 21 per cent. He would delay operation in cases of discrete but complicated peritonitis. Furbringer joins with him in deprecating indiscriminate surgical treatment, and records a mortality of 7·4 per cent. in adults and only 3·7 in children. A very large number of the latter will, he thinks, recover completely without operation. Reclus insists upon a connection between entero-colitis and appendicitis,[13] and mentions an instance where a patient gave herself a poisonous enema, causing severe inflammation of the colon. After this subsided it was followed by an attack of appendicitis, Thus he would include an ascending appendicitis among the varieties of the disease. A hysterical causation is also admitted in a few cases by Talamon. The bacteria of the disease, according to Achard and Broca,[14] include not only the bacillus coli, but also the streptococcus and pneumococcus, either alone or with the B. coli. Possibly other organisms may take an active part at times in the process, and it seems clear that occasionally the bacillus coli is entirely absent.

1 B. Med. Jour., May 1. 2 Glasgow Med. Jour., Ap. 3 B. Med. Jour., Mar. 13. 4 Therap. Gaz., Feb. 18. 5 B. Med. J., Jan. 23. 6 Ibidem. 7 Practitioner, May. 8 Int. Med. Magaz., March. 9 Med. Week, Mar. 6. 10 Med. Week, Mar. 19. 11 B. Med. Jour, May 1. 12 Medical Week, Mar. 5. 13 Med. Week, Ap. 2. 14 Ibidem.

---

### DISEASES OF THE LUNGS.—TUBERCULOSIS.

(*Continued from page* 216.)

**Paquin's Serum.**—B. McG., aged 18 years and two months, had been suffering with joint and bone tuberculosis for seven years, and had had ten operations performed on different parts of his body to open abscesses, and to remove necrosed bone. The seat of the primary trouble was the right hip joint, but was giving him trouble on every limb. The left tibia was much involved, having at one time eight openings discharging what was called a tuberculous pus. The hip had three openings that would heal and open alternately, and one that was open continually for seven years. He had an abscess on each arm, one of the sternum, one of the index finger of the right hand, a tubercular nodule in the skin of the scrotum, an abscess near the apex of the left scapula, and two or three on the lower jaw. He was without medical aid for two years. In the fall of 1895 he was put in care of Dr. G. W. Broome, of St. Louis, who performed an operation to remove necrosed bone from the thigh and tibia, thinking that these openings would heal. This having failed, he decided to try the serum treatment, and was taken in charge by Dr. G. W. Cale, of St. Louis. He began the treatment in March, 1895, at which time he had four abscesses discharging mixed pus, and two others that afterward opened. He had daily injections of serum in doses of 23 to 30 minims, and at the close of six months' treatment five of these abscesses had closed; he had gained ten pounds, and was without temperature. All of the abscesses are now closed and have been for months, with the exception of a very slight one on the thigh, and a piece of dead bone, to be soon removed, has been located there, which is the cause of its remaining open. Up to the present time he has gained 16 pounds, has a good appetite, is enjoying good general health, and works daily. In every one of these cases the diagnosis was based on careful physical examination and microscopical analysis.

**Tuberculocidin**, we are told by Ernst Klebs, contains, in addition to the substance found in antiphthisin, certain constituents of the tubercle bacilli which render the preparations even more effective as a protective agency. It is given in the same way as antiphthisin, but it will generally be found necessary to give smaller doses, as it more readily produces a rise of temperature. At the commencement of any course of treatment, and especially in the case of sensitive patients, it is more advisable to inject antiphthisin. On the other hand, tuberculocidin is especially suitable for subsequent repetition of the treatment. The total quantity necessary for a single course of treatment is from 50 to 200 c. cm. The treatment must be repeated once or twice annually for several successive years, both in those cases where relapses occur, and in those where the patient is apparently cured.

The treatment with antiphthisin or tuberculocidin should only be commenced when the sputum is free, or nearly free, from any admixture with organisms. Under the circumstances the amount of irritation produced in any given case by these bacteriacidal measures must be ascertained, and the doses increased accordingly, either quickly or slowly. By the adoption of this method the patient will gradually become accustomed to the treatment, and the best possible results obtained. Klebs states that, as a general rule, a moderate reaction is most desirable and healthy; whereas, reactions accompanied by high fever and unfavourable symptoms should be avoided, and the injections should be at once diminished, and for a time entirely discontinued.

**Surgical Treatment of Lung Disease.**—We do not propose to enter here into the surgical treatment of pleuritic disease, empyema, &c., but rather to review some recent contributions on the surgery of the lung itself, and this only as far as it is a matter of practical interest to the physician who should at least be acquainted with the resources of surgical art in relieving some of the conditions hitherto relegated to the medical as distinguished from the surgical practitioner. A comprehensive article by G. Ryerson Fowler,[14] of Brooklyn, on "The Surgery of Intrathoracic Tuberculosis" reviews the whole question as regards tuberculous disease. Passing over the first portion dealing with empyemata, pyopneumothorax and other affections of the pleural cavity, we find he discusses the direct treatment of tuberculous cavities. He refers to Mosler the credit of having made the first systematic attempts to introduce remedies from without through the thoracic wall into lung cavities, although such proposal had been made by Bary in 1726, and subsequently by Nare, Herff, and Hooken.

Although Mosler's attempts were without ultimate benefit to the patients, yet they served to demonstrate that systematic treatment of pulmonary cavities is possible. Hillier (1894) attempted in three instances the use of direct injections of solution of corrosive sublimate into the parenchyma of the lungs. The quantity employed did not exceed ten centigrammes at one injection, yet in all three cases the effects upon the patients were such as to compel him to abandon the treatment.

Passing on to the question of pneumotomy, Fowler states that surgical operations with the view of reaching and treating cavities in the lungs can only be indicated in cases in which circumscribed pulmonary affections exist, and the patient's strength will admit of the interference. Great difficulty must necessarily be experienced in the selection of proper cases because of the uncertainty of determining the limits of the disease, even at the hands of those skilled in physical diagnosis.

Cases in which an apparently isolated tuberculous affection of the lung exists and which is progressing but slowly only can be considered proper for trial of surgical methods. But to be certain that the disease is stationary, or but slowly progressive, is not in itself an easy matter. To operate in a case in which the disease has come to a standstill would be an unjustifiable interference, for the reason that it is this class of cases that undergoes cure by natural processes. Again, the presence or absence of adhesions between the costal and pulmonary layers of the pleura is of importance. While these occur in a certain proportion of cases of extensive cavities superficially situated, failure in this respect will greatly complicate the post-operative course of the case. Further, the exact location of the cavity itself is an important consideration. But a limited area of the pulmonary tissue is accessible to the surgeon. In the majority of cases the proper field for operation will be found to be bounded in an upward direction by the clavicle, inward by the manubrium sterni, externally by the pectoralis minor muscle, and downward by the second rib.

Bronchiectatic cavities can still less be the objects of successful operative attacks. These are usually multiple, and the difficulties of exactly locating the same greatly increased. Fowler cites, however, Sonnenburg's one successful case.

In summing up the indications for pneumotomy, Mauclaise limits the operation in tuberculous cases to those cavities that are circumscribed and without peripheral tuberculous infiltration, and to certain cavities of doubtful nature which form at the expense of both pleura and lung—viz. pulmonary abscesses, consecutive to tuberculous spondylitis and tuberculous caries of the ribs. Fowler cites three examples of this class of cases.

A certain class of cases known as tuberculous abscesses have been reported as healed by direct incision. These abscesses of the lung are not necessarily tuberculous in their origin. When they do thus originate they are probably small foci of circumscribed tuberculous deposit, which become infected with the bacteria of suppuration. It is more than likely that many of these cases are not tuberculous in their origin. The usual course followed in reaching a cavity in the area indicated is as follows. The relations of the first rib to the clavicle are first made out. The incision is made parallel with the rib, and includes the muscular coverings of the chest wall, as well as its tendino-pericostal investments and the fibres of the intercostal muscles. The periosteum of the rib is separated, particularly at its posterior surface, in order to avoid injury of the intercostal artery. The remainder of the intercostal muscles are separated from the pleura, thus freeing the entire intercostal space. In order to gain more space the lower edge of the first rib, when the latter is not covered by the clavicle, may be gnawed away with a rongeur forceps. Resection of a rib is scarcely possible in this locality. In the further steps of the operation it is important to remember that, upon the right side the innominate vein passes downward behind the sterno-clavicular articulation, the articulation of the second rib corresponding with the middle of the superior vena cava. Upon the left side the vein commences immediately below the cartilage of the first rib close to the sternum, and descends almost vertically, in its course describing a slight curve, the convexity of which is towards the right. Behind this the arterial trunks arising from the aorta are found. The subclavian artery lies transversely in front of and upon a somewhat lower level than the apex of the lung. The pleura is reflected from the rounded pulmonary apex directly to the concave margin of the first rib.

The lung tissue is divided with the Paquelin cautery knife heated to a dull red. The progress of the instrument in the direction of the cavity should be slow, in which case the hæmorrhage is but slight. The surgeon should be satisfied if he succeeds in entering the cavity. He should not attempt to explore it in its entire extent, the wound of entrance becoming gradually dilated subsequently by retamponning with gauze. The absence of sensibility of the pulmonary tissue is a striking feature, and for this reason after the pleura is incised no anæsthetic is necessary.

[14] Annals of Surgery, Nov., 1896.

